# Can Immunoexpression of Cancer Stem Cell Markers Prognosticate Tongue Squamous Cell Carcinoma? A Systematic Review and Meta-Analysis

**DOI:** 10.3390/jcm12082753

**Published:** 2023-04-07

**Authors:** Sayantanee Chaudhury, Swagatika Panda, Neeta Mohanty, Saurav Panda, Diksha Mohapatra, Ravishankar Nagaraja, Alkananda Sahoo, Divya Gopinath, Natalia Lewkowicz, Barbara Lapinska

**Affiliations:** 1 Department of Oral Pathology and Microbiology, Institute of Dental Sciences, Siksha ‘O’ Anusandhan, Deemed to be University, Bhubaneswar 751003, India; 2 Department of Periodontics and Implantology, Institute of Dental Sciences, Siksha ‘O’ Anusandhan, Deemed to be University, Bhubaneswar 751003, India; 3 Department of Biostatistics, Vallabhbhai Patel Chest Institute, University of Delhi, Delhi 110021, India; 4 Basic Medical and Dental Sciences Department, Ajman University, Ajman P.O. Box 346, United Arab Emirates; 5 Centre for Transdisciplinary Research, Saveetha Dental College, Saveetha Institute of Medical and Technical Science, Chennai 600077, India; 6 Department of Periodontology and Oral Diseases, Medical University of Lodz, 251 Pomorska St, 92-213 Lodz, Poland; 7 Department of General Dentistry, Medical University of Lodz, 251 Pomorska St, 92-213 Lodz, Poland

**Keywords:** cancer stem cell markers, tongue squamous cell carcinoma, survival rate, clinicopathological, oral squamous cell carcinoma

## Abstract

The objective was to evaluate the association of the immunoexpression of cancer stem cell (CSC) markers with clinicopathological and survival outcomes in tongue squamous cell carcinoma (TSCC) patients. This systematic review and meta-analysis [PROSPERO (CRD42021226791)] included observational studies that compared the association of clinicopathological and survival outcomes with CSC immunoexpression in TSCC patients. Pooled odds ratios (ORs) and hazard ratios (HRs) with 95% confidence intervals (CI) were used as outcome measures. Six studies identified the association with three surface markers (c-MET, STAT3, CD44) and four transcription markers (NANOG, OCT4, BMI, SOX2). The odds of early-stage presentation were 41% (OR = 0.59, 95% CI 0.42–0.83) and 75% (OR = 0.25; 95% CI 0.14–0.45) lower in CSC and SOX2 immuno-positive cases than immuno-negative cases, respectively. The odds of well-differentiated tumors in transcription marker immuno-positive cases were 45% lower compared to immuno-negative cases (OR = 0.55, 95% CI 0.32–0.96). The odds of positive lymph nodes were 2.01 times higher in CSC immuno-positive cases compared to immuno-negative cases (OR = 2.01, 95% CI 1.11–3.65). Mortality in immuno-positive cases was 121% higher than that in immuno-negative cases (HR = 2.21; 95% CI 1.16–4.21). Advanced tumor staging and grading, lymph node metastasis, and mortality were significantly associated with positive immunoexpression of CSC markers.

## 1. Introduction

Oral squamous cell carcinomas (OSCC) are diverse and complex malignancies exhibiting high inter and intra-tumor heterogeneity [1], which impedes therapeutic success and dictates individual-specific adverse outcomes. This poses a challenge in identifying objective prognostic and predictive biomarkers. The last few decades have evidenced tremendous advancement in foraging several components of the tumor microenvironment which can predict nodal metastasis and treatment outcomes. These components of the tumor microenvironment include tumor-associated macrophages (TAM), tumor-associated endothelial cells (TAE), tumor-infiltrating lymphocytes (TIL), cancer-associated fibroblasts (CAF), and cancer stem cells (CSCs). Due to the indefinite self-renewal ability to initiate and maintain tumor growth, a small population of CSCs has been put forward as a significant determinant in intra-tumor heterogeneity [2,3]. After the identification of leukemia stem cells [4], the role of CSCs was also identified in various solid tumors [5,6,7], including OSCC [8,9,10]. Resistance to therapeutics and tumor recapitulating potential confer CSCs the candidacy for determining survival outcomes. ALDH1A1, CD44, BMI1, OCT4, SOX2, and CD133 are the widely used CSC markers in head and neck squamous cell carcinoma [11]. SOX-8, a member of the Sry-like high-mobility group box (SOX) genes family regulates cancer stem cell-like properties and cisplatin-induced epithelial-mesenchymal resistance in tongue squamous cell carcinoma by acting on the Wnt/β catenin pathway [12]. There are divisive remarks on the predictive potential of CSCs in OSCC [13,14,15,16,17,18]. While many authors have evidenced the prognostic potential of the CSCs [8,9,10,14,17,18], few have contradicted so [19]. Studies have shown negative [14,18,20,21] and positive [8,9,10,17] associations of CSCs with clinicopathologic features and survival outcomes of OSCC such as recurrence, lymph node metastasis, distant metastasis, and second primary. Because the sub-sites in the oral mucosa influence the survival outcomes differently [18,20,22,23], the absence of site-specificity in the experiments may have added to these discordant results. The tongue is one of the most common sites of OSCC and is known to harbor stem cells that often are intrinsically resistant to chemo-radiotherapy leading to relapse. We hypothesize that the immunoexpression of CSCs in the resected specimen may have a role in predicting survival outcomes in tongue squamous cell carcinoma (TSCC) and hence can be regarded as a putative prognostic factor. Thus, the objective of this systematic review and meta-analysis was to evaluate the association of the immunoexpression of CSC biomarkers with clinicopathological features and outcomes in resected TSCC.

## 2. Materials and Methods

This systematic review abided by the Preferred Reporting for Systematic Review and Meta-analysis (PRISMA) checklist as described in systematic reviews and meta-analyses [24]. This systematic review and meta-analysis were registered in the PROSPERO database and received the registration number CRD42021226791.

### 2.1. Study Design

This systematic review and meta-analysis of human studies included observational cross-sectional studies, which compared the association of clinicopathological features and outcomes with CSC biomarker immuno-positive and CSC biomarker immuno-negative cases of resected TSCC.

### 2.2. Search Strategy

Both MESH and keywords were used to search in PUBMED and SCOPUS. We used the following key phrases: (“Neoplastic stem cells” OR “cancer stem cells” OR “tumor stem cell” OR CSC) AND (tongue OR Oral) AND (carcinoma OR cancer OR malignancy OR neoplasm OR tumor) AND (immunohistochemistry OR “immunohistochemical markers” OR immunoexpression OR biomarkers OR OCT4 OR NANOG OR SOX2 OR STAT3 OR BMI-1 OR CD44 OR CD133 OR c-MET OR ALDH-1) AND (prognosis OR prognoses OR outcome OR survival OR recurrence OR nodal status OR “lymph node metastasis” OR “distant metastasis” OR “second primary”). The search was carried out until 28 February 2022 without limits. The reference lists of the selected articles and narrative reviews were also searched to exclude the possibility of missing a study.

### 2.3. Study Selection

Two investigators (SC, SP) independently screened the identified articles initially by title and abstract, followed by full texts considering the inclusion and exclusion criteria. The third investigator (NM) resolved the disagreements between these two investigators. Observational cross-sectional studies associating the immunoexpression status of CSC biomarkers with the clinicopathologic features and outcomes in resected TSCC specimens were included. Those studies which have reported one or multiple outcomes such as distant metastasis, overall survival, disease-free survival, recurrence, and second primary, in the number or percentage of patients who underwent surgery as the primary treatment modality, were included. Studies demonstrating the hazard ratio (HR) in the Kaplan–Meier curve were excluded. Studies in languages other than English and studies where authors have used isoform of any CSC markers were excluded. The guidelines from the Reporting Recommendations for Tumor Marker Prognostic Studies (REMARK) [25,26] were used to qualify the included studies.

### 2.4. Data Extraction

Three investigators (SP, SC, DG) independently extracted information such as first author name, year of publication, population studied, the primary antibody used, sample size, age, male-to-female ratio, tumor size, nodal status, grading, follow-up period, and the number of patients reported with one or multiple survival outcomes in both immuno-positive and immuno-negative TSCC patients. Because there was no uniformity in reporting TNM staging, we combined T1 and T2 as the early stage and T3 and T4 as the advanced stage of presentation of the tumor. For the same reason, we have pooled moderate and poor histopathological grading as one.

The association of immunoexpression of CSC markers with quantitative values of clinico-pathologic features such as tumor size, histopathological grading, and lymph node metastasis was subjected to meta-analysis in Revman (version 5.4.1) to find the odd’s ratio (OR) with 95% confidence interval (CI). The association of CSC immunoexpression with survival outcomes was expressed as the Hazard ratio (HR) with 95% CI. Forest plots with random effects were constructed for each reported outcome with the respective OR/HR. Sub-group analysis was planned for subgroups of CSCs such as transcription and surface markers as well as for similar markers. Sensitivity analysis was conducted by removing one study at one point in time. *p*-values were two-sided with a significance value of less than 0.05. The methodological bias of all the included studies was assessed according to the Newcastle Ottawa Scale (NOS) checklist for cross-sectional studies [27]. Disagreements were resolved by discussion among authors. Publication bias was assessed by Egger’s test.

## 3. Results

### 3.1. Study Selection

**Figure 1:**
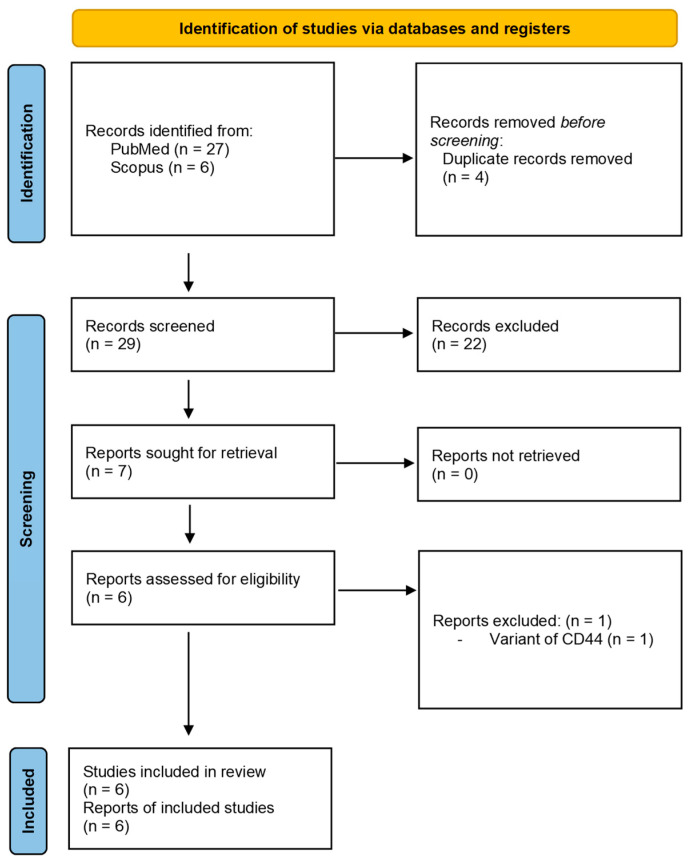
PRISMA flowchart showing the study selection process.

A total of thirty-three records were selected in two databases in the first phase. After removing the duplicate studies, there were twenty-nine articles which, upon a further comprehensive evaluation of titles and abstracts, shortlisted only seven articles. The full text of all selected articles was evaluated, thereby excluding one article because of the inclusion of a variant of the CD44 rather than the normal form [28]. No citations were identified during the grey literature search. Only six articles were considered for the second phase evaluation [26,29,30,31,32,33]. Four CSC markers such as B lymphoma Mo-MLV insertion region 1 (BMI), Nanog homeobox (NANOG), Octamer-binding Transcription Factor-4 (OCT4), and Cluster of Differentiation 44 (CD44) have been studied by Rodrigues et al. [34]. Tyrosine-protein kinase Met (c-MET) has been studied by two authors [29,30], and Sex Determining Region Y HMG-box 2 (SOX2) has been investigated by another two authors [31,33]. Signal Transducer and Activator of Transcription 3 (STAT3) was investigated in a single study [32]. Figure 1 presents the PRISMA flow chart.

### 3.2. Study Characteristics

We have identified six observational cross-sectional studies [29,30,31,32,33,34] associating the immunoexpression of seven CSC markers (c-MET, NANOG, OCT4, BMI, SOX2, STAT3, CD44) with clinicopathological features and outcomes (Table 1 and Table 2). Out of the seven CSC markers, there were three surface markers [29,30,31], and the rest were transcription markers [31,32,33,34]. These studies were conducted in the Korean [29,30], Brazilian [34], Chinese [31,32,33], and Japanese [32] populations. All the studies were based on a small cohort of fewer than 100 subjects. Gender-wise distribution of TSCC cases was reported in all studies except the study conducted by Rodrigues et al. [34]. A higher prevalence of TSCC was observed in males compared to females. The age range of participants could not be determined because of the differences in reporting. Tumor size was reported in all the studies [29,30,31,32,33,34], where the early stage of presentation of TSCC was more frequent compared to the advanced stage. Grading was reported in only five studies [29,30,31,32,33], where the prevalence of moderate to poor grading was observed [31,32,33]. The association of immunoexpression of seven [29,30,31,32,33,34] CSC markers was reported with lymph node metastasis and five [31,34] with the overall survival of the patients. Furthermore, two [30,31] studies reported recurrence and distant metastasis. The odds of developing recurrence and distant metastasis in immuno-positive cases were found to be 5.41 (95% CI 1.35–21.67, *p* = 0.02) and 5.54 (95% CI 1.69–18.15, *p* = 0.004) times higher compared to immuno-negative cases as pooled from Kim et al. [30] and Du et al. [31], respectively.

### 3.3. Quality Assessment

The Newcastle Ottawa (NOS) Scale 25 for the cross-sectional study was used to evaluate the quality of included studies based on three parameters such as selection, comparability, and outcome. The NOS score of each study was found to be nine, indicating the inclusion of high-quality studies (Table 1).

### 3.4. Meta-Analysis

#### 3.4.1. Association of CSC Markers with Tumor Stage

**Figure 2:**
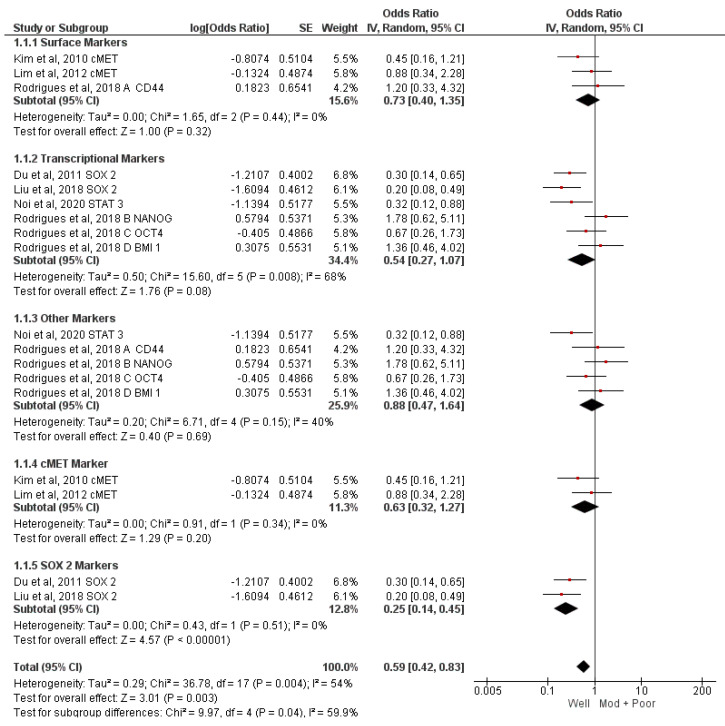
Meta−analysis of CSC markers and tumor stage [29,30,31,32,33,34].

Meta-analysis of all the markers together revealed that the odds of early-stage presentation in immuno-positive cases are 41% lower compared to immuno-negative cases (OR = 0.59, 95% CI 0.42–0.83; *p* = 0.003, I^2^ = 54%). Subgroup analysis was conducted to evaluate the association of c-MET [27,28] and SOX2 [29,31] with the tumor stage, which showed that the odds of early-stage presentation in SOX2 immuno-positive cases are 75% lower compared to immuno-negative cases (OR = 0.25; 95% CI 0.14–0.45, *p* < 0.00001, I^2^ = 0%). Surface markers and transcription markers were studied in three [29,30,34] and six articles [31,32,33,34], respectively, for which a subgroup analysis was conducted. None of the two was found to be significantly associated with the tumor stage. These findings are represented in Figure 2.

#### 3.4.2. Association of CSC Markers with Histological Grading

**Figure 3:**
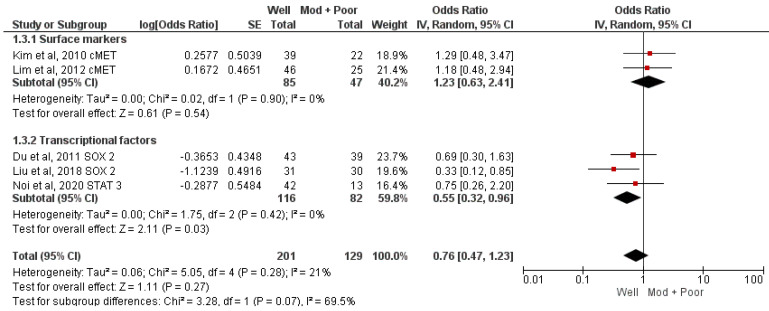
Meta-analysis of CSC markers and histological grading [29,30,31,32,33].

Five studies analyzed the association of three CSC markers with histopathological grading [29,30,31,32,33]. No significant association (well vs. moderate and poor; OR = 0.76, 95% CI 0.54–1.09; *p* = 0.13 I^2^ = 21%) was found in the meta-analysis (Figure 3). Subgroup analysis for identifying the association of c-MET [29,30] and SOX2 [31,32,33] with histological grading did not reveal any significant association. Surface markers were analyzed in two studies [29,30] and transcription markers in three studies [31,32,33], which concluded that the odds of well-differentiated tumors in transcription marker-positive cases were 45% lower compared to transcription marker negative cases (OR = 0.55, 95% CI 0.32–0.96, *p* = 0.03, I^2^ = 0%) (Figure 3).

#### 3.4.3. Correlation of CSC Markers with Nodal Metastasis

**Figure 4:**
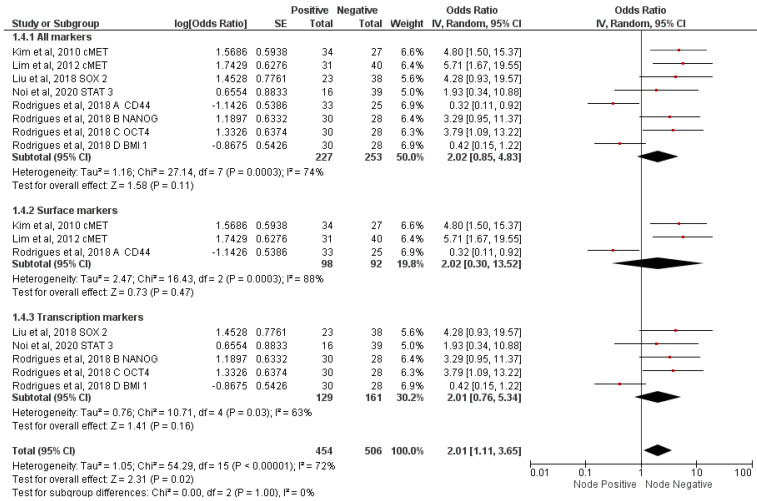
Meta−analysis of CSC markers and nodal metastasis [29,30,32,33,34].

Five studies analyzed the association of seven CSC markers with nodal metastasis [29,30,32,33,34]. Meta-analysis of all markers demonstrated a significant association (OR = 2.01; 95% CI 1.11–3.65; *p* = 0.02; I^2^ = 72%). There were three articles each for surface markers [29,30,34] and transcription markers [32,33,34]. Subgroup meta-analysis conducted on surface and transcription markers demonstrated no significant association (Figure 4).

#### 3.4.4. Correlation of CSC Markers with Overall Survival

**Figure 5:**
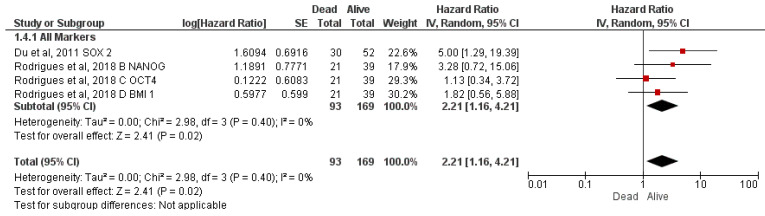
Correlation of CSC markers with overall survival [31,34].

There were only two studies [31,34] that reported the association of four CSC markers with overall survival (Figure 5). Mortality in immuno-positive cases was 121% higher than that in immuno-negative cases (HR = 2.21; 95% CI 1.16–4.21; *p* = 0.02; Heterogeneity I^2^ = 0%).

### 3.5. Heterogeneity Analysis

The heterogeneity was substantial and moderate in associating CSC immunoexpression with nodal metastasis (72%) and tumor size (54%), respectively. The heterogeneity was the least (0%) while evaluating the association of transcriptional CSC markers with grading, SOX2 immunoexpression with tumor size, and CSC/transcription markers immunoexpression with overall survival.

### 3.6. Sensitivity Analysis

In the sensitivity analysis, the influence of each study on the pooled OR and HR was examined by repeating the meta-analysis while omitting each study, one at a time. None of the analyses showed major deviation after the removal of each study except the analysis of nodal metastasis from the study conducted by Rodrigues et al. [34]. As Rodrigues et al. evaluated four markers in their one study such as CD44 (A), NANOG (B), OCT4 (C), and BMI1 (D), A and D together brought down the heterogeneity to 0%. This makes the meta-analysis results robust and reliable.

### 3.7. Publication Bias

Egger’s test was performed to evaluate the publication bias of the studies that were included in the meta-analyses, which concluded that no publication bias in these meta-analyses existed (Table 3).

## 4. Discussion

In addition to the other components of the tumor microenvironment such as TAM, TAE, TIL, and CAF, cancer stem cell markers can be considered the appropriate candidates to be explored for their prognostic potential because of their capacity to initiate and sustain neoplastic changes. A very recent meta-analysis has suggested the prognostic potential of CSC markers, particularly CD133, NANOG, and OCT4, in head and neck squamous cell carcinoma [35]. However, the prognostic potential of CSC biomarkers is site-specific, and the precision can be increased by focusing on specific subsites in the oral cavity [36]. Therefore, this manuscript addressed the current lack of consensus on the association of immunoexpression of CSC biomarkers with clinicopathologic features and overall survival in TSCC. Moreover, CSC biomarkers can be broadly divided into surface and transcription markers which were duly considered in this meta-analysis by conducting a subgroup analysis. Among the markers studied, c-MET and CD44 are the surface markers whereas SOX2, STAT3, OCT4, NANOG, and BMI1 are the transcription markers.

Easy accessibility and interpretability of immunohistochemistry are the reasons to elucidate the prognostic potential of CSC immunoexpression. A systematic search with set inclusion criteria resulted in the selection of six studies comparing the association of clinicopathologic features and mortality between immuno-positive and immuno-negative TSCC cases. The available studies were conducted in East Asian populations such as Japan [32], China [31,32,33], and Korea [29,30], with only one study conducted in Brazil [34]. Studies have revealed a higher male prevalence and predominance of early-stage presentation of TSCC. Tumor size and histopathologic grading were the clinical and pathological factors, respectively, which were subjected to meta-analysis. The association between SOX2 immunoexpression and advanced tumor size was found to be significant (OR = 0.25; 95% CI 0.14–0.45, *p* < 0.00001, I^2^ = 0%). The tumor size was also found to be significantly associated (*p* = 0.003) with the overall immunoexpression of CSCs, although the heterogeneity among studies was 54%. Similar findings have been reported by Ma et al. [37], who studied oropharyngeal tumor samples and found a positive association of immunoexpression of SOX2 and OCT4 with tumor size. Although c-MET expression was not significantly associated with tumor size, Lim et al. [29] demonstrated a significant association with more than a 4 mm depth of tumor invasion which eventually shortens the patient survival time. Similarly, histopathological grading was found to be significantly associated with immunoexpression of transcriptional CSC markers such as SOX2 and STAT3 (OR = 0.55; 95% CI 0.32–0.96, *p* = 0.03, I^2^ = 0%). Previous studies also demonstrated that expression levels of OCT4, NANOG, and SOX2 were higher in poorly differentiated tumors than that of well-differentiated breast cancers, glioblastomas, and bladder carcinomas [37] as well as OSCC [17]. It can be concluded that more than surface markers, transcriptional markers pose a significant role in tumor progression.

The odds of having nodal metastases in CSC biomarker immuno-positive cases were found to be 2.01 times higher compared to that of immuno-negative cases (OR = 2.01, 95% CI 1.11–3.65, *p* = 0.02, I^2^ = 72%). This finding paves the way for exploring the lymphangiogenic potential of CSCs. Evidence of CSCs promoting local angiogenesis and endothelial progenitor cell mobilization by stimulating pro-angiogenic factors such as VEGF and SDF-1 may further support this possibility as both angiogenesis and lymphangiogenesis involve similar growth factors [38]. Ma et al. [37] have also shown the positive association of OCT4 and SOX2 with lymph node metastasis in oropharyngeal squamous cell carcinoma. Wakamatsu et al. [39] have identified the association between CSC markers and nodal metastasis in gastric cancer.

The CSC biomarkers associated with OS, qualified for the meta-analysis, were all transcriptional markers. The risk of mortality was found to be 2.21 times high in immunopositive cases compared to that in immuno-negative cases (HR = 2.21, 95% CI 1.16–4.21; *p* = 0.02, I^2^ = 0%). This finding was similar to the recent meta-analysis demonstrating that high expression of CSC biomarkers, particularly CD133, OCT4, and NANOG, was associated with poor OS [35]. Immunoexpression of transcriptional markers, particularly SOX2, was reportedly associated with the progression of colorectal carcinoma [40], head and neck cancer, and OSCC [17,41,42,43]. A remarkable prognostic potential of SOX2 in carcinoma of the lung, breast, colon, hepatic, and head and neck SCC was also suggested in a meta-analysis conducted by Javaeed et al. [44]. Two studies [45,46] have also concluded a negative association of immunoexpression of CSC markers with prognostic outcomes. Due to the absence of any study, this systematic review shed no light upon the possible association of CSC immunoexpression with the second primary cancer. Therefore, there is a need for research on CSC immunoexpression in second primary OSCC. There are only two studies, each reporting the association of immunoexpression of CSC markers with recurrence and distant metastasis [30,31]. The odds of developing recurrence and distant metastasis in immuno-positive cases were found to be 5.41 (95% CI 1.35–21.67, *p* = 0.02) and 5.54 (95% CI 1.69–18.15, *p* = 0.004) times higher compared to immuno-negative cases as pooled from Kim et al. [30] and Du et al. [31], respectively.

The studies included in the meta-analysis were not functionally identical because of the differences in methodologies and differences in effect size, for which the random effect model was applied. The heterogeneity in all analyses was insignificant except for nodal metastasis, which remains unexplained except for the possibility of inconsistency among the studies.

There are several limitations of this systematic review and meta-analysis. First, the number of included studies, as well as the sample size in each study, was relatively small, which might have reduced the power and accuracy of the meta-analysis. Second, nodal metastasis and overall survival are dependent upon unadjusted OR and RR, respectively. Therefore, outcome analysis would have been more consistent with matched analysis by adjusting multiple factors like age, TNM staging, histopathological grading, treatment modalities, and follow-up time. Third, differences in the cut-off values for the immuno-positive cases might have increased the methodological heterogeneity.

Although two studies [45,46] have denied the correlation of immunoexpression of CSC markers with prognostic outcomes, the majority of studies have consistently found a positive correlation [17,40,47]. Therefore, it can be concluded that CSC markers may facilitate the acquisition of growth potential in selected tumor cells. This also supports the cancer stem cell model, which proposes that only a subset of cells in the tumor microenvironment has tumor-initiating potential. Contradicting the conventional ‘stochastic model’ of carcinogenesis, the findings of this study have evidenced the fact that biologically distinct subsets of cells within the tumor microenvironment might have the potential not only to initiate but also to progress the tumor. The findings of this systematic review and meta-analysis have paved the way for developing CSC-targeted therapies in TSCC.

Taken together, we suggest that the immunoexpression of CSC biomarkers, particularly SOX2 alone or in combination with other transcription markers, can behave as a consistent prognostic determinant. Translating this research finding into clinical practice requires further prospective large well-defined research on the prognostic ability of the individual as well as combined markers. This would not only facilitate decision-making by the surgeon but also practice the appropriate follow-up protocol in TSCC.

## 5. Conclusions

Within the limitations of this study, we can conclude that TSCC might represent a cancer stem cell model based on the significant association of the positive immunoexpression of CSC markers with advanced tumor size, advanced histopathological grading, lymph-node metastasis, and high mortality. Therefore, the IHC of CSC markers may be considered a potential prognostic tool in TSCC. Further prospective studies using standardized immunohistochemical interpretation techniques and matched analysis for possible confounders would help strengthen the present evidence, which would probably make the immunohistochemical evaluation of CSC markers a regular prognostic assessment strategy for every patient. The prospective study can also address the differences in immunohistochemistry results caused by antibody specificity, tissue collection, preservation, and archiving of the formalin-fixed paraffin-embedded tissues.

## Figures and Tables

**Table 1:** Study characteristics.

Author	IHC Marker	Population	N	Age	M:F	Follow Up (years)	Cut-Off Value	Compliance to REMARK Guidelines	NOS Score
Lim et al., 2012 [29]	c-MET	Korea	71	NM	50:21	6.1	>30%	Study design was incompletely described	9
Kim et al., 2010 [30]	c-MET	Korea	61	22–79	41:20	11.6	≥30%	Study design was incompletely described	9
Rodrigues et al., 2018 [34]	OCT4	Brazil	60	≥40	NM	5	25–49%	fulfilled	9
Rodrigues et al., 2018 [34]	BMI1	Brazil	60	≥40	NM	15	25–49%	fulfilled	9
Rodrigues et al., 2018 [34]	NANOG	Brazil	60	≥40	NM	5	25–49%	fulfilled	9
Noi et al., 2020 [32]	STAT3	Japan	63	33–86	34:29	8	NM	Study design was incompletely described	9
Du et al., 2011 [31]	SOX2	China	82	NM	55:27	6	25%	fulfilled	9
Liu et al., 2018 [33]	SOX2	China	61	53.4	35:26	10	NM	Study design was incompletely described	9
Rodrigues et al., 2018 [34]	CD44	Brazil	60	≥40	NM	5	25–49%	fulfilled	9

IHC = immunohistochemical; N = sample size; M:F = male to female ratio; NM = not mentioned; NOS = Newcastle-Ottawa Scale.

**Table 2:** Study characteristics: expression of cancer stem cells markers vs. clinicopathological features and outcomes.

Author	IHC Marker	Immuno-Positive Cases	TNM Stage				Grading		Outcome in Immuno-Positive Cases				Immuno-Negative Cases	TNM Stage				Grading		Outcome in Immuno-Negative Cases			
			t1 + t2	t3 + t4	Nodes (+)	Nodes (−)	Well	Moderate to Poor	LNM	Recurrence	DM	OS		t1 + t2	t3 + t4	nodes (+)	nodes (−)	Well	Moderate to Poor	LNM	Recurrence	DM	OS
Lim et al., 2012 [29]	c-MET	39	17	22	24	15	24	12	24	NM	NM	NM	32	15	17	7	25	22	13	7	NM	NM	NM
Kim et al., 2010 [30]	c-MET	33	26	7	24	9	22	11	24	13	2	NM	28	25	3	10	18	17	11	10	13	2	NM
Rodrigues et al., 2018 [34]	OCT4	46	24	12	23	13	NM	NM	23	NM	NM	23	24	18	6	7	15	NM	NM	7	NM	NM	16
Rodrigues et al., 2018 [34]	BMI 1	23	17	6	8	13	NM	NM	8	NM	NM	13	37	25	12	22	15	NM	NM	22	NM	NM	26
Rodrigues et al., 2018 [34]	NANOG	39	29	10	23	14	NM	NM	23	NM	NM	22	21	13	8	7	14	NM	NM	7	NM	NM	17
Noi et al., 2020 [32]	STAT 3	40	27	13	13	27	30	10	13	NM	NM	NM	15	13	2	3	12	12	3	3	NM	NM	NM
Du et al., 2011 [31]	SOX 2	51	31	20	0	51	25	26	0	20	12	26	31	26	5	0	31	18	13	0	20	12	26
Liu et al., 2018 [33]	SOX 2	39	26	13	19	20	16	23	19	NM	NM	NM	22	20	2	4	18	15	7	4	NM	NM	NM
Rodrigues et al., 2018 [34]	CD 44	51	36	15	27	23	NM	NM	27	NM	NM	30	9	6	3	6	2	NM	NM	6	NM	NM	9

IHC = immunohistochemical; NM = not mentioned; TNM = tumor, nodes, metastasis; LNM = lymph node, metastasis; DM = Distant metastasis; OS = overall survival.

**Table 3:** Egger’s test to evaluate the publication bias.

Clinicopathological Parameters	Coefficient	Estimate with 95% CI	*p*-Value
Tumor stage	Slope	−4.3754 (−8.1679, 0.5829)	0.059
	Bias	7.6302 (−0.0431, 15.2172)	0.051
Grading	Slope	−0.2267 (−11.7652, 11.3117)	0.954
	Bias	−0.0892 (−23.9120, 23.7335)	0.991
Nodal status	Slope	−3.4001 (−7.2616, 0.4614)	0.080
	Bias	6.3674 (−0.2440, 12.4907)	0.073
Overall survival	Slope	−3.2114 (−14.7502, 8.3273)	0.354
	Bias	6.1181 (−11.4157, 23.6519)	0.272

## Data Availability

Not applicable.
